# Increased Prevalence of Glycoprotein IIb/IIIa Leu 33 Pro Polymorphism in End Stage Renal Disease Patients on Hemodialysis

**Published:** 2008-09

**Authors:** Amein Al-Ali, Fahad Al-Muhanna, Samir Al-Mueilo, Emmanuel Larbi, Ali Al-Sultan, Abdullah Rubaish, Suad Al-Ateeq, Alhusain Al-Zaharani

**Affiliations:** 1 *Departments of Biochemistry, College of Medicine, King Faisal University, Saudi Arabia;*; 2 *Department of Internal Medicine, College of Medicine, King Faisal University, Saudi Arabia*

**Keywords:** hemodialysis, fibrinogen receptor, polymorphism, glycoprotein, platelets, CVD

## Abstract

Traditional atherosclerosis risk factors cannot elucidate the increased prevalence of cardiovascular events in end stage renal disease (ESRD) patients on hemodialysis. A previous study has indicated a strong association of the PI^A1/A2^ polymorphism with myocardial infarction, diabetes and renal allograft rejection. In this investigation, we determined the prevalence of the PI^A1/A2^ polymorphism of platelet glycoprotein (GP) IIb/IIIa in ESRD patients on hemodialysis in the Eastern Province of Saudi Arabia. The PI^A1/A2^ polymorphism was determined in 42 ESRD patients receiving hemodialysis and in 49 subjects without current or past history of renal disease. Genotypes were determined by a reverse-hybridization assay and were confirmed by restriction fragment length polymorphism procedures. The PI^A2^ allele frequency among the control sample was 28.6% (2 were homozygous for PI^A2^, 23 were homozygous for PI^A1^, and 24 were heterozygous PI^A1/A2^). The PI^A2^ allele frequency among the hemodialysis sample was 50% (2 were homozygous for PI^A2^, 2 were homozygous for PI^A1^ and 38 were heterozygous for PI^A1/A2^). The PI^A2^ allele frequency among the hemodialysis patients was significantly higher than that in the control group [Odds ratios 2.5 (1.35-4.61), *p*<0.003; Adjusted odds ratios of 2.21 (1.05-4.65), *p*<0.036 after adjustment for the presence of diabetes; Simultaneously adjusting the odds ratios for the presence of standard risk factors (diabetes and hypertension) gave an adjusted OR of 6.87 (1.54-30.71), *p*=0.064]. These results suggest that the PI^A2^ polymorphism may contribute toward the etiology of cardiovascular diseases in ESRD patients. A further study with a larger sample size is needed to confirm above results.

## INTRODUCTION

The most abundant platelet surface receptor is the platelet glycoprotein (GP) IIb/IIIa, which binds to fibrinogen and Willebrand factor (1-2). This plays a central role in platelet aggregation and adhesion to subendothelial tissues, which is an essential first step in thrombus formation (3-4). The gene encoding GP IIb/IIIa is subject to considerable genetic polymorphism. The expression level and structure of the platelet GP receptor are influenced by these polymorphisms. The PI^A1/A2^ polymorphism, which is due to a T to C substitution at position 1565 in exon 2 of the GPIIb/IIIa gene, is common in many populations. This missense mutation leads to the substitution of leucine (PI^A1^) with proline (PI^A2^) (5). Many studies have reported a strong association between PI^A2^ polymorphism and myocardial infarction, diabetes mellitus and renal allograft rejection (6-8).

Traditional CVD risk factors are common in dialysis patients even at the initiation of dialysis therapy (9). However, the increased rate of morbidity and mortality in these patients cannot be explained solely by the presence of these factors. Genetic factors such as a single nucleotide polymorphism, which are the most common form of DNA variation in the human genome, may significantly influence the severity of disease in these patients (10-12). Hence, our attention was directed towards the study of the presence of genetic factors such as the GP IIb/IIIa polymorphism in these patients that may influence the rate of increased risk for CVD. This will help in the identification of interindividual variability in risks for the disease.

## METHODS

A case-control study was conducted in 42 ESRD patients on hemodialysis admitted to the hemodialysis unit of King Fahad Hospital of the University, Al-Khobar, Saudi Arabia. Normal subjects matched with case patients for age and gender were recruited from the general population. The sole exclusion criterion for control subjects was past, present or family history of renal disease. Informed consent was obtained from both patients and the control group. Blood samples were collected in EDTA tubes and were frozen until analysis. Genomic DNA was obtained from 300ul whole blood using QIAamp Blood Kit (Qiagen, Hilden, Germany) according to the manufacturer’s protocol. Genotypes were determined by a reverse-hybridization assay developed by Vienna Laboratory (13). In this method, the relevant gene sequences are simultaneously invitro amplified and biotin labeled in a single amplification reaction. The amplified products are selectively hybridized to a test strip that contains allele specific oligonucleotide probes immobilized as an array of parallel lines. The results were routinely confirmed by restriction fragment length polymorphism (RFLP) (7). The procedures encompassed the amplification of exon two by polymerase chain reaction (PCR) using the primers 5’- ttc tga ttg ctg gac ttc tct t – 3’ (sense) and 5’-tct ctc ccc atg gca aag agt – 3’ (antisense). Exon two amplificate carrying the mutation can be restricted by Nci I creating two restriction fragments (216, 50 bp) and by Msp I resulting in three restriction fragments (171, 50, 45 bp). The wild type allele does not contain comparable restriction sites.

Genotype and allele frequencies were estimated by gene counting and expressed as percentages of the total. The χ^2^ test was used to compare differences between groups. Difference was considered statistically significant when *p*<0.05. To study association and adjust for confounders a logistic regression analysis was carried out. Odds ratios (OR) are given with 95% CI).

## RESULTS

A total of 91 subjects (42 patients and 49 controls) were analyzed. Base line characteristics of the study groups are shown in Table 1. The prevalence of diabetes, hypertension and history of coronary artery disease in the hemodialysis patients was significantly higher than that in the controls. The age and gender of the two groups were similar. The duration of time the patients had been on hemodialysis ranged between 1–121 months. The PI^A2^ allele frequency among the control sample was 28.6% (2 were homozygous for PI^A2^, 23 were homozygous for PI^A1^ and 24 were heterozygous for PI^A1/A2^) (Table 2). These values are slightly higher than those found in other populations (14-16). The PI^A2^ allele frequency among the hemodialysis sample was 50% (2 were homozygous for PI^A2^, 2 were homozygous for PI^A1^ and 38 were heterozygous for PI^A1/A2^) (Table 2). The PI^A2^ allele frequency among the hemodialysis patients was significantly higher than that in the control. [Odds ratios 2.5 (1.35-4.61), *p*<0.003; Adjusted odds ratios of 2.21 (1.05-4.65), *p*<0.036 after adjustment for the presence of diabetes; simultaneously adjusting the odds ratios for the presence of standard risk factors (diabetes and hypertension) gave an adjusted OR of 6.87 (1.54-30.71), *p*=0.064]. The Exact Test was used to determine whether the observed genotype conformed to Hardy Weinberg equilibrium (HWE) expectations. The p values were 0.29 and 1.491 × 10^-7^ in the control and patient groups respectively. These values suggest that the genotypic proportions follow HWE expected proportions in the control group, but not in the patient group. However such deviations have been seen in small sample size studies and may suggest the presence of an association (17, 18). Within each study group, there was no significant association between genotype and cardiovascular risks such as age, hypertension or the duration of hemodialysis.

**Table 1:** Baseline characteristics of ESRD patients and control subjects

	Controls	Patients

Number	49	42
Age, mean ± SD (Years)	51.6 ± 13.6	49.8 ± 16.51
Gender (Male)	61%	68%
Hypertension	10%^a^	77% (*p*<0.001)
Diabetes	5%^a^	52.8% (*p*<0.001)
CVD	–	33%
Duration of Dialysis (Months)	–	Range 1-121

a In the general population.

**Table 2:** Allele frequency and genotype distribution of PI^A1/A2^ gene polymorphism of platelet glycoprotein GP IIb/IIIa in patients on hemodialysis and control group

Genotype	Control		Patients		Odd ratio	Adjusted Odd ratio^a^
	frequency	%	frequency	%		

**PI**^A1/A1^	23	46.9	2	4.8	7.5 (2.44-23.17) *p*<0.0001	4.94 (1.47-16.61) *p*=0.01^a^
**PI**^A1/A2^	24	49	38	90		6.87 (1.54-30.71) *p*=0.012†
**PI**^A2/A2^	2	4.1	2	4.8		
**Allele:**						
**PI**^A1^	70	71.4	28	28.6	2.5 (1.35-4.61) *p*=0.003	2.21 (1.05-4.65) *p*=0.036^a^
**PI**^A2^	42	50	42	50		2.35 (0.95-5.84) *p*=0.064†

a After adjustment for presence of diabetes.

## DISCUSSION

Cardiovascular risk factors are highly prevalent in end stage renal disease patients receiving dialysis therapy and accounts for over 20% of child and adult mortality (19). Moreover multiple coagulation abnormalities are also commonly present in these patients, including decreased responsiveness of platelets to stimulation (20), though the increased prevalence of cardiovascular events in these patients cannot be attributable to these factors alone. Single nucleotide polymorphism has recently drawn attention toward its role in influencing the prevalence of vascular calcification or the etiology of the disease in these patients (21). GP PI^A2^ polymorphism has been associated with increased platelet aggregability which in turn increases the risk of myocardial infarction or stroke (22). However, the clinical role of PI^A2^ as a risk factor for cardiovascular disease or diabetic nephropathy is still controversial. (11, 23-26). Many studies have shown no such association between this polymorphism and an increased risk of myocardial infarction (11, 23). More recently, Tschoepe et al. has shown a strong association between this polymorphism and diabetes mellitus (7). In addition, Salido et al. has shown an association between the PI^A2^ polymorphism of glycoprotein IIIa and the occurrence of renal allograft acute rejection during the first post transplant year (8). Gawaz et al. has concluded that the platelets of patients with chronic renal failure reveal an aggregation defect that is at least partially due to an intrinsic GP IIb/IIIa dysfunction (27).

This is the first study to report the prevalence of this polymorphism in dialysis patients in the Eastern Province of Saudi Arabia. The allelic prevalence of PI^A2^ among our control group was determined to be 28.2% which is higher than that reported for European populations (12). However, in a study carried out in Taiwan, it was found that this polymorphism was absent in their population (16).

In our study, the allelic prevalence of PI^A2^ polymorphism among the end stage renal disease patients receiving dialysis was significantly higher than that in the control group with an OR of 2.5 which indicates that it is two-fold higher than in the control group. However, the significance for the association with the polymorphism was lost (modest) in sub-groups analyzed by diabetes and hypertension as confounding factors. It has to be emphasized that the majority of patients in this study were diabetic, which may confirm the strong association of this polymorphism with diabetes as reported earlier (7). However, this needs to be confirmed by a further study with a larger sample size. In any case, this study may infer that this allelic polymorphism has some pathogenic impact on the course of renal failure, and it may provide nephrologists with a more precise approach for the identification of high-risk ESRD patients.
